# Supplementary material for: Direct RNA sequencing of the Escherichia coli epitranscriptome uncovers alterations under heat stress
Source: Nucleic Acids Res. 2025 Mar 20;53(6):gkaf175. doi: 10.1093/nar/gkaf175 (PMC11925731; doi:10.1093/nar/gkaf175)
Supplement: gkaf175_Supplemental_File [file gkaf175_supplemental_file.pdf]

Direct RNA sequencing of the *Escherichia coli* epitranscriptome uncovers alterations under heat stress

Sebastian Riquelme Barrios[1]; Leonardo Vasquez Camus[1]; Siobhan A. Cusack[1]; Korinna Burdack[1], Dimitar Plamenov Petrov[1], G. Nur Yeşiltaç-Tosun[2]; Stefanie Kaiser[2]; Pascal Giehr[3]*; Kirsten Jung[1]*

[1]Faculty of Biology, Microbiology, Ludwig-Maximilians-Universität München, 82152 Martinsried, Germany

[2]Goethe University Frankfurt, Faculty 14, Institute of Pharmaceutical Chemistry, 60438 Frankfurt

[3]Department of Chemistry, Ludwig-Maximilians-Universität München, 81377 München, Germany

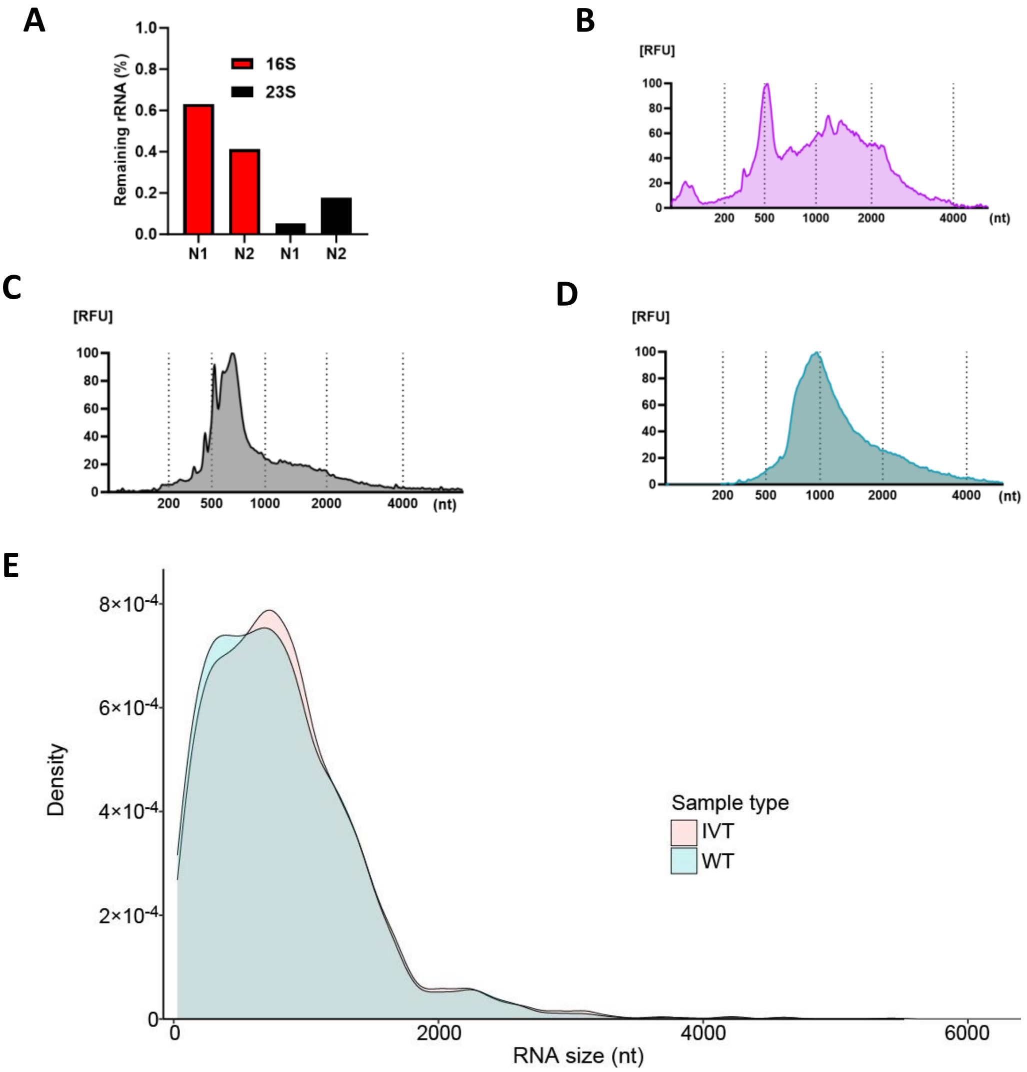

Supplementary Figure 1. Quality control of prepared RNA samples. (A) qRT-PCR was used to determine the abundance of 16S rRNA (red) and 23S rRNA (black) remaining after tRNA and rRNA depletion of WT RNA. rRNA abundance was normalized to that of the *recA* transcript. N1 and N2 indicate biological replicate samples. (B–D) Electropherograms showing relative fluorescent units (RFUs) at each nucleotide (nt) position in (B) tRNA/rRNA-depleted and polyadenylated WT samples, (C) *in vitro* transcribed (IVT) *E. coli* RNA samples before polyadenylation, and (D) IVT *E. coli* RNA after polyadenylation. (E) Size distribution of RNAs that were present at a depth of < 20 reads in the WT and IVT samples. There was no statistically significant difference in mean RNA size between the two sample types ($p > 0.05$; Student's *t*-test).

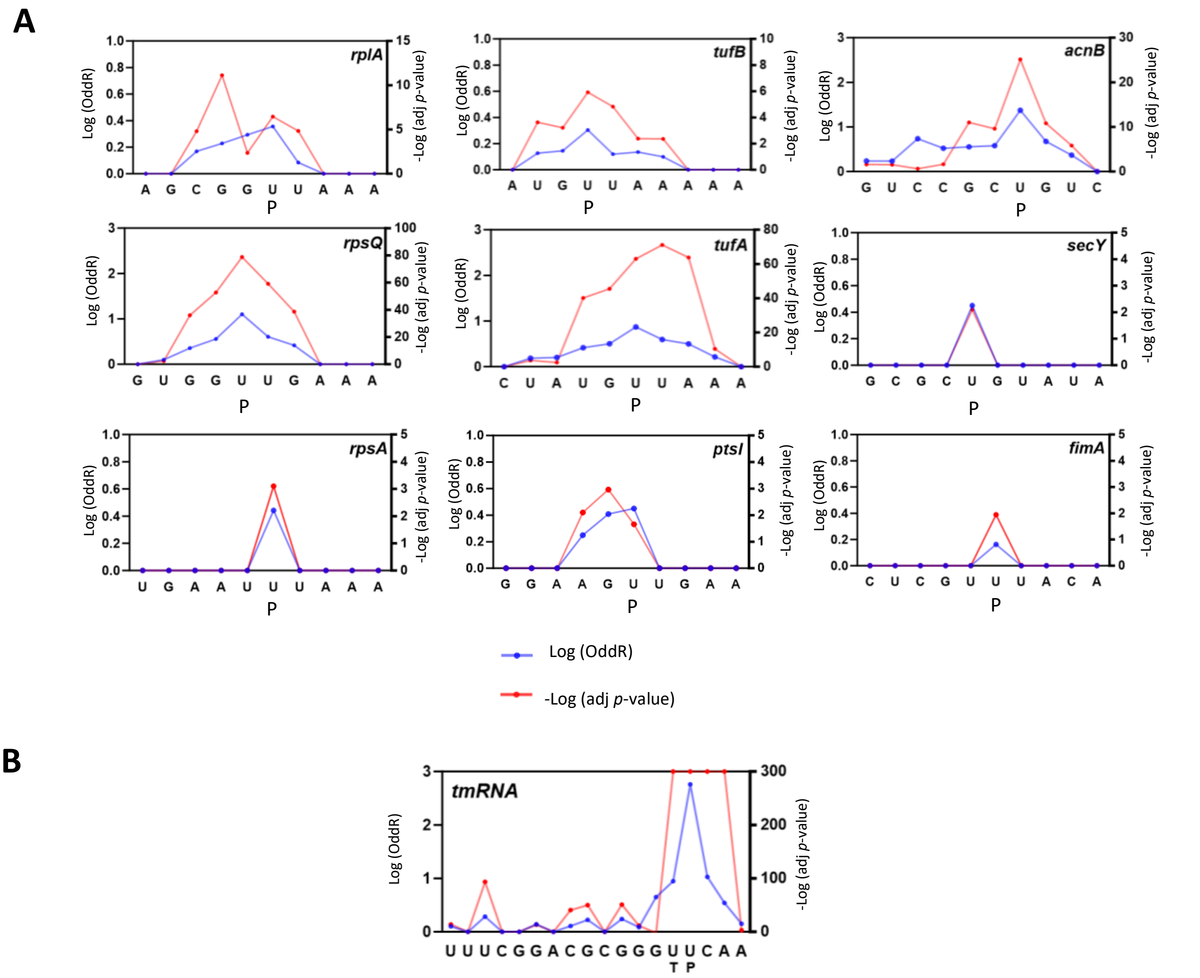

Supplementary Figure 2. Detection of known RNA modifications in mRNA and transfer-messenger RNA (tmRNA). (A, B) Log-transformed odds ratio (oddR) and negative log-transformed adjusted *p*-values for (A) nine sites previously identified as pseudouridine (P) in the *E. coli* mRNA [18] and (B) positions 341 and 342 of the *E. coli* tmRNA *ssrA*. T, 5-methyluridine (m^5U).

A

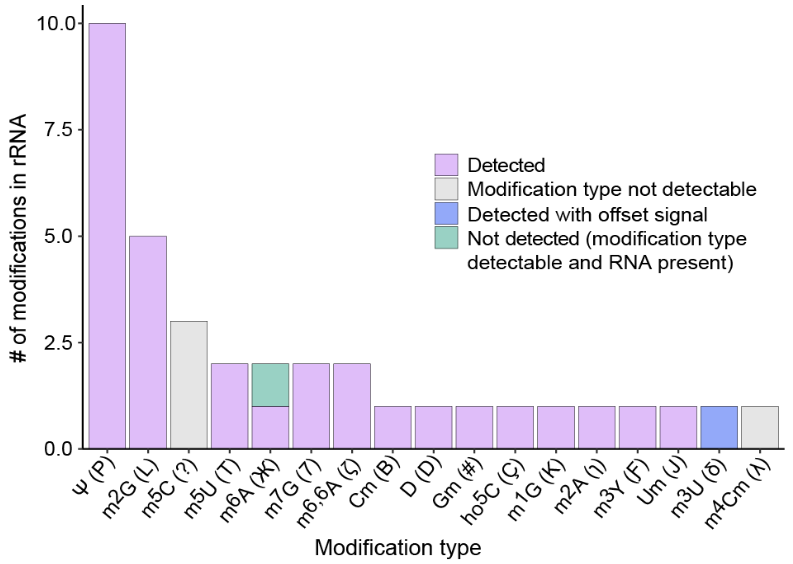

B

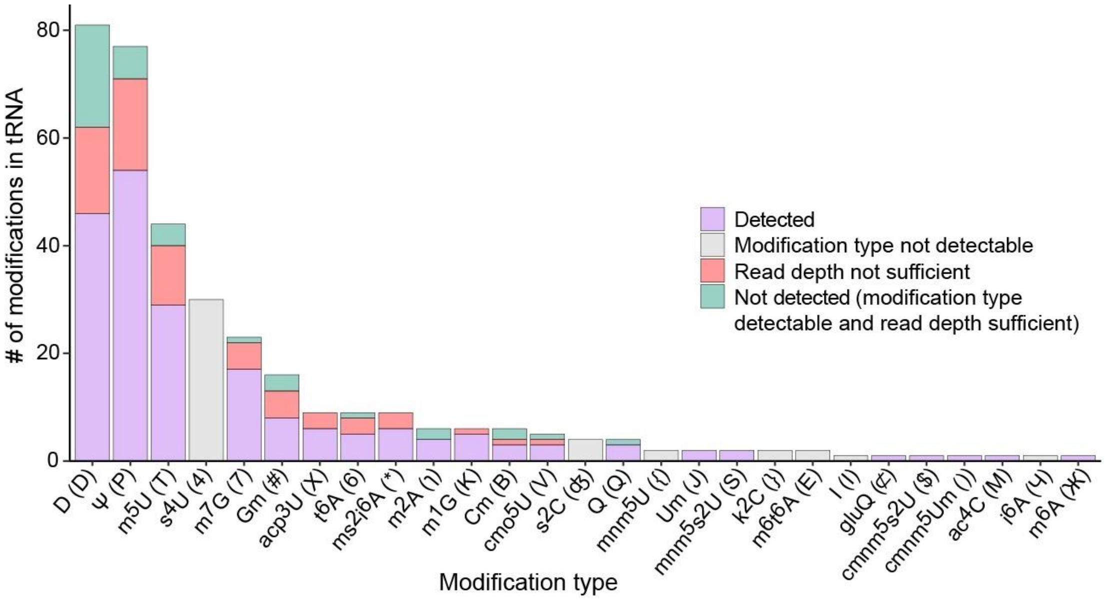

Supplementary Figure 3. Detection of known modifications in *E. coli* tRNA and rRNA with ELIGOS. The presence of known modifications in the ELIGOS output was evaluated for *E. coli* (A) rRNA and (B) tRNA. Modifications were classified as detected; of a modification type not known to be detectable with ELIGOS; contained in an RNA that was not present at a sufficient sequencing depth (20 reads); or of a modification type that was detectable with ELIGOS and contained in an RNA that was present at a sufficient sequencing depth in our dataset but not detected.

A

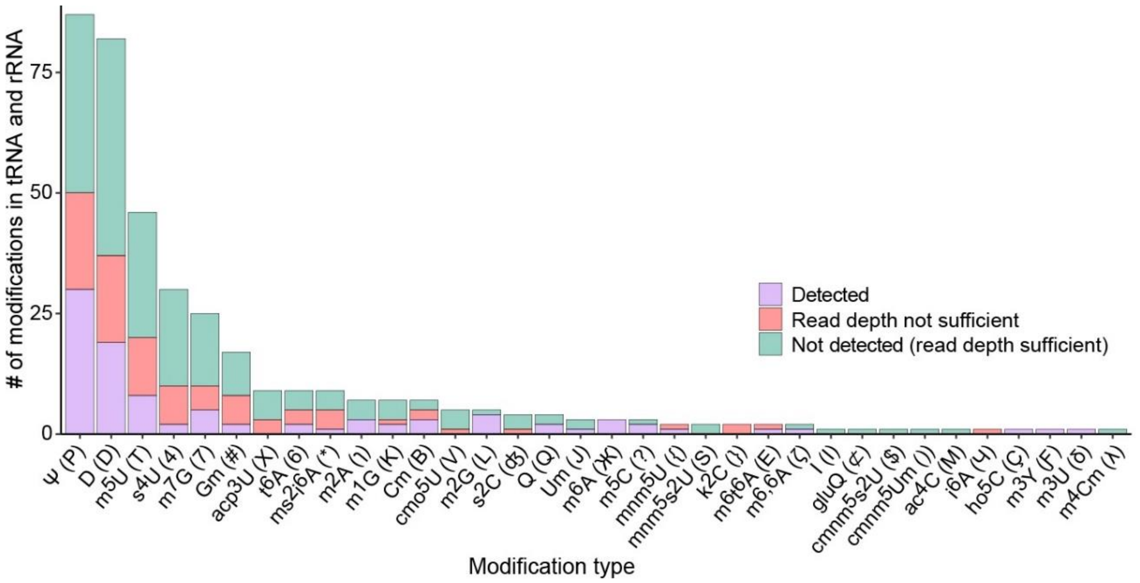

B

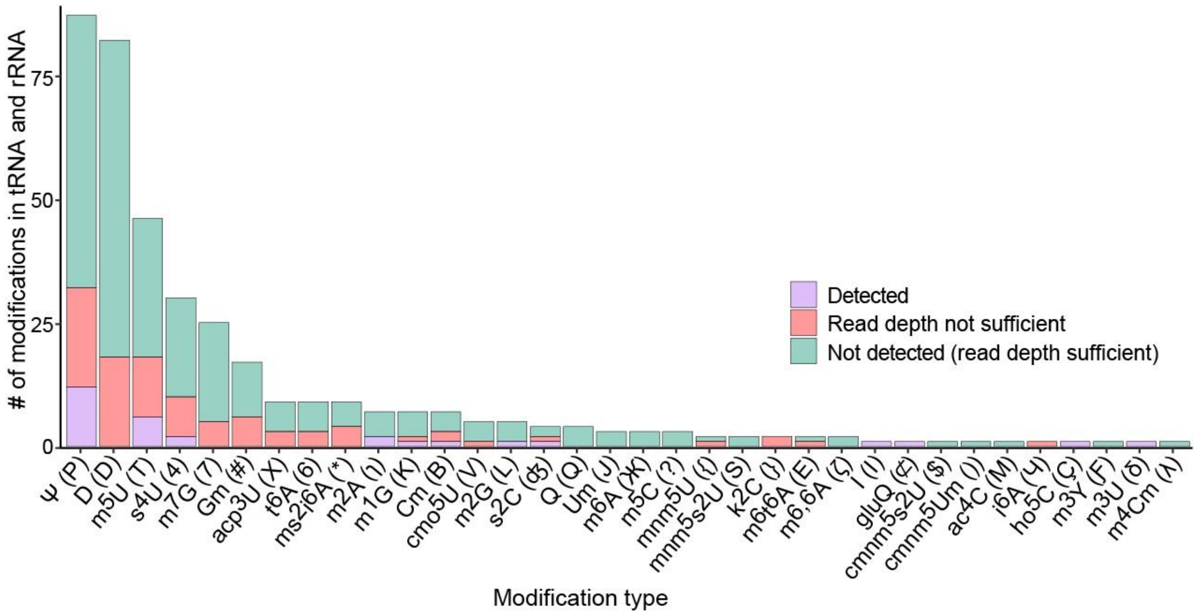

Supplementary Figure 4. Detection of known modifications in *E. coli* tRNA and rRNA with Nanocompore and DRUMMER. (A, B) Detection of known modifications in the *E. coli* tRNA and rRNA with (A) Nanocompore and (B) DRUMMER. Modifications were classified as detected; contained in an RNA that was not present at a sufficient sequencing depth (20 reads); or contained in an RNA that was present at a sufficient sequencing depth in our dataset but was not detected.

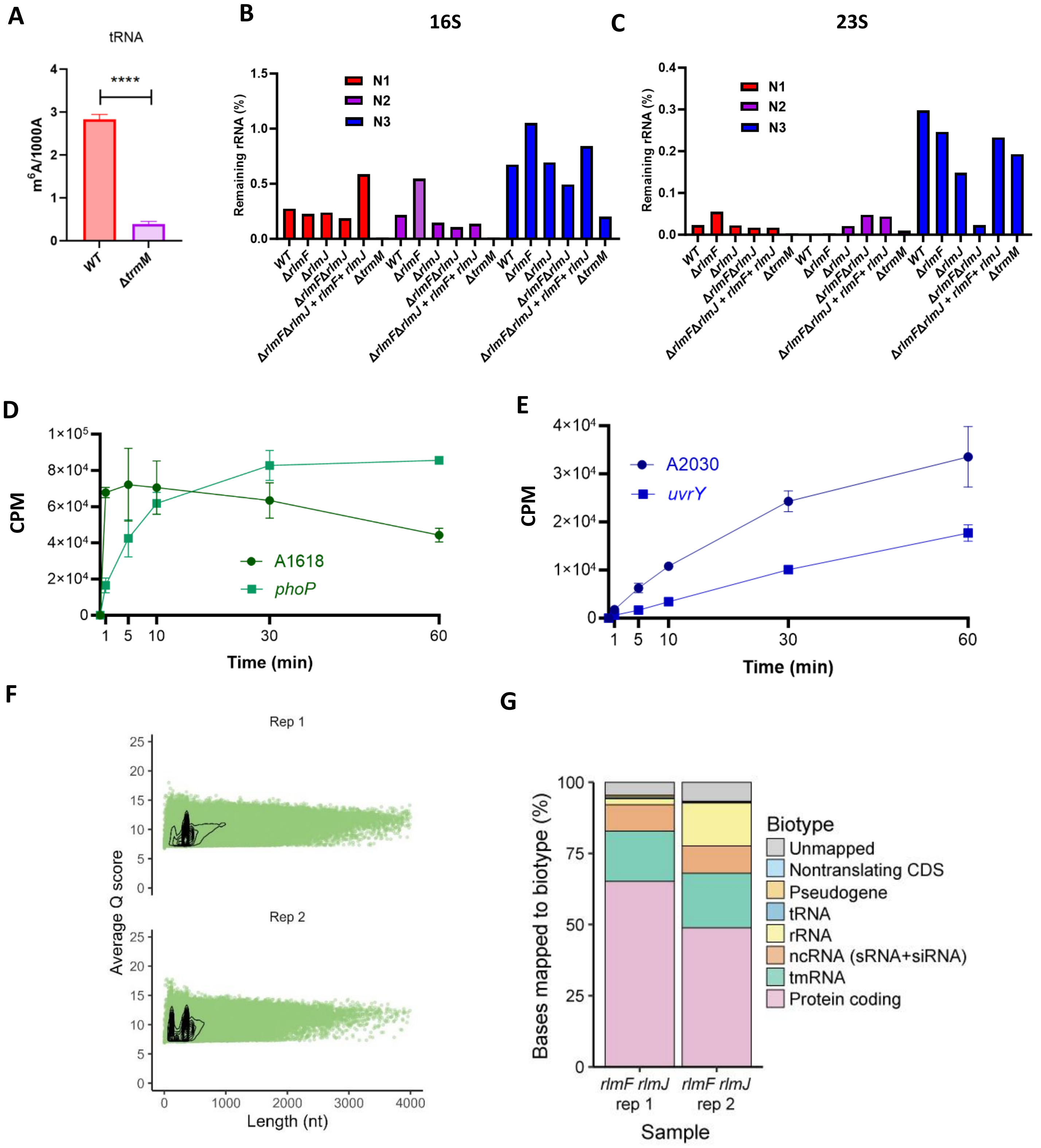

A
tRNA
m6A/1000A
****
WT
ΔtrmM
B
16S
Remaining rRNA (%)
N1
N2
N3
WT
ΔrlmF
ΔrlmJ
ΔrlmFΔrlmJ
ΔrlmFΔrlmJ + rlmF+ rlmJ
ΔtrmM
C
23S
Remaining rRNA (%)
D
CPM
A1618
phoP
Time (min)
E
CPM
A2030
uvrY
Time (min)
F
Rep 1
Rep 2
Average Q score
Length (nt)
G
Bases mapped to biotype (%)
Biotype
Unmapped
Nontranslating CDS
Pseudogene
tRNA
rRNA
ncRNA (sRNA+siRNA)
tmRNA
Protein coding
rlmF rlmJ rep 1
rlmF rlmJ rep 2
Sample

Supplementary Figure 5. Detection of m^6A in WT and methyltransferase mutant *E. coli* RNA. (A) Mass spectrometric measurements of tRNA samples collected from WT and Δ*trmM* mutant *E. coli* samples. Data are presented as the number of modified As (m^6A) per 1000 unmodified As (1000A). ****p < 0.0001 (unpaired Student's *t*-test). (B, C) Percentages of (B) 16S and (C) 23S rRNA remaining after rRNA depletion in three biological replicates of several *E. coli* strains: WT; the single mutants Δ*rlmF*, Δ*rlmJ*, and Δ*trmM*; the double mutant Δ*rlmF*Δ*rlmJ*; and the complementation strain Δ*rlmF*Δ*rlmJ* + *rlmF* + *rlmJ*. qRT-PCR was performed to calculate the percentage of remaining rRNA (16S or 23S rRNA) in each sample, normalized to *recA* transcript abundance. N1–N3 indicate biological replicate samples. (D, E) Methyltransferase activities of (D) RlmF and (E) RlmJ over the course of 1 h. IVT RNAs of 100–300 nt in size were generated for *phoP* and *uvrY*, each of which contained an expected modification site; 30-nt RNAs containing As in the appropriate sequence context were also generated for the 23S rRNA positions 2030 and 1618. At time zero, the reaction was started by adding ^3H-methionine. CPM, counts per minute. (F, G) Summary of sequencing data generated from two biological replicates of Δ*rlmF*Δ*rlmJ* *E. coli* RNA, namely (F) Q score per read vs. read length and (G) relative proportions of bases mapping to specific RNA types. tmRNA, transfer-messenger RNA; ncRNA, noncoding RNA; sRNA, small RNA; siRNA, small interfering RNA; CDS, coding sequence.

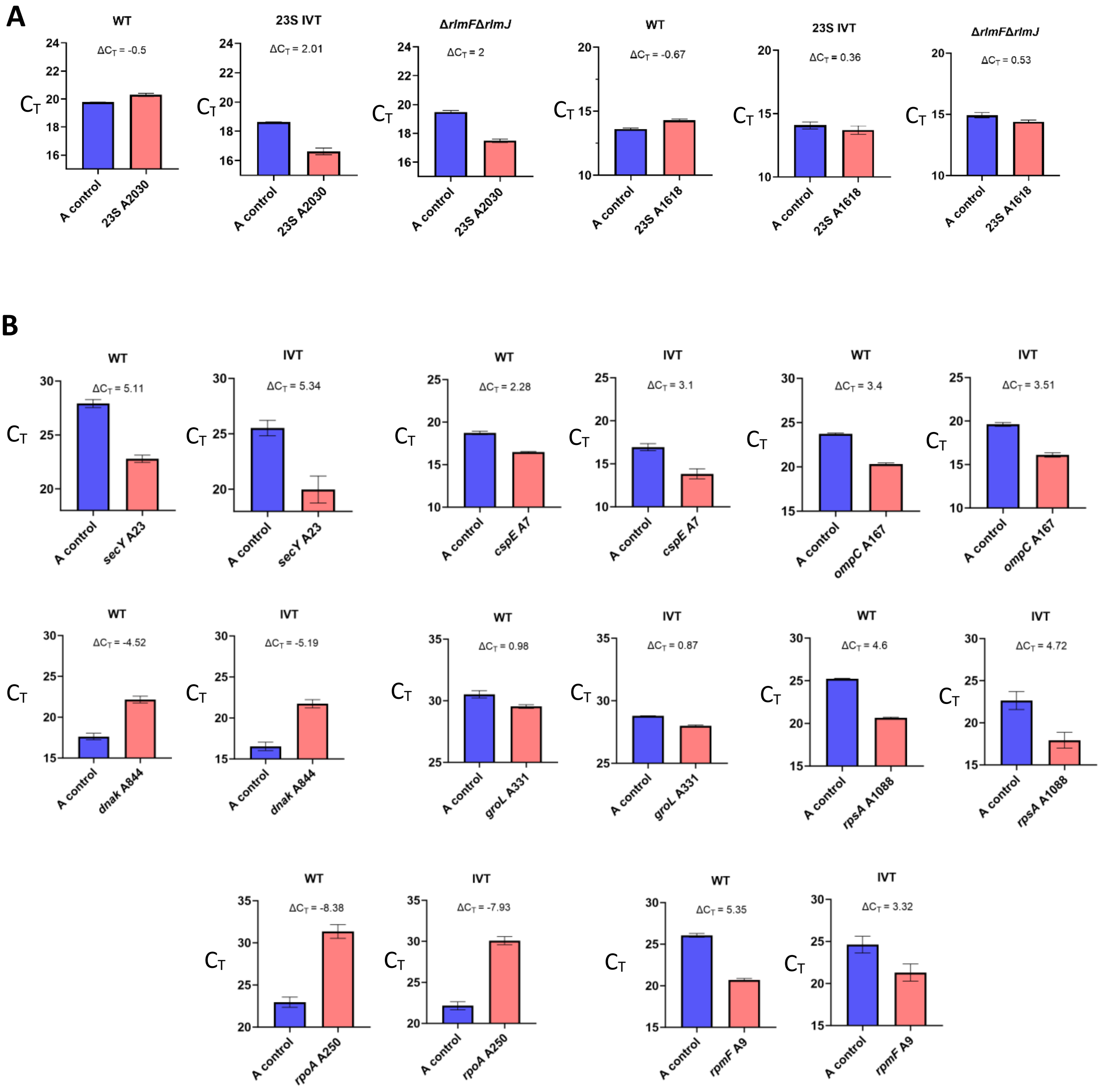

Supplementary Figure 6. m^6A detection with SELECT. (A) Detection of m^6A modifications at the A2030 and A1618 sites of the 23S rRNA in WT, IVT, and Δ*rlmF*Δ*rlmJ* *E. coli* with SELECT. (B) Assessment of eight *E. coli* mRNA sites previously identified as m^6A modifications [21] with SELECT. For each target m^6A site, a nearby unmodified control sequence was also tested (marked "A control"). Data are presented as the mean ± standard deviation from three biological replicates.

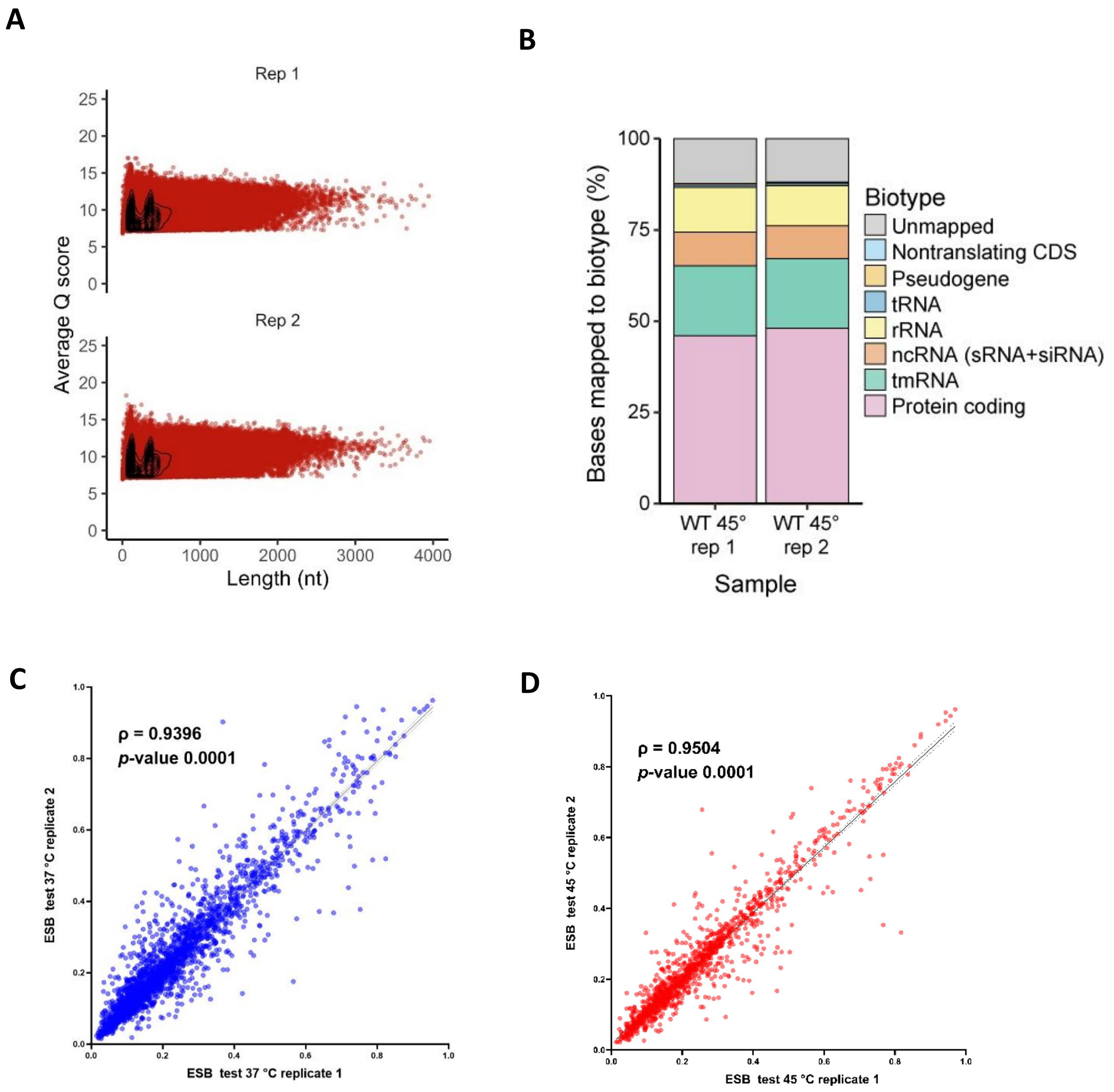

Supplementary Figure 7. Summary of direct RNA sequencing data generated from *E. coli* exposed to heat stress. (A, B) Summary of sequencing data generated from two biological replicates of WT *E. coli* grown at 45 °C, namely (A) Q score per read vs. read length and (B) relative proportions of bases mapping to specific RNA types. (C, D) Correlations between ESB test values from two biological replicates each (C) grown under standard growth conditions (37 °C) and (D) exposed to heat stress (45 °C). Correlations were calculated as Pearson's correlation coefficient (ρ).

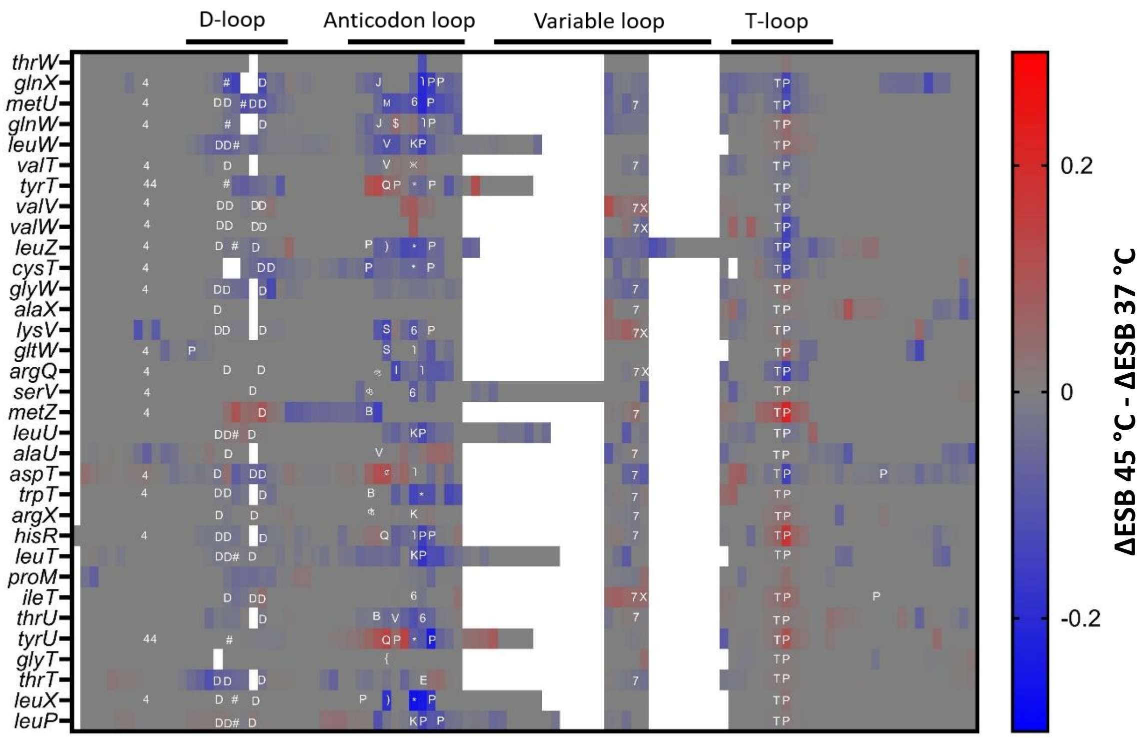

Supplementary Figure 8. Changes in *E. coli* tRNA modification abundance in response to heat stress. Changes in tRNA abundance were calculated as the ΔESB at 45 °C - ΔESB at 37 °C for positions across the 33 tRNAs containing modifications that were detected with ELIGOS.

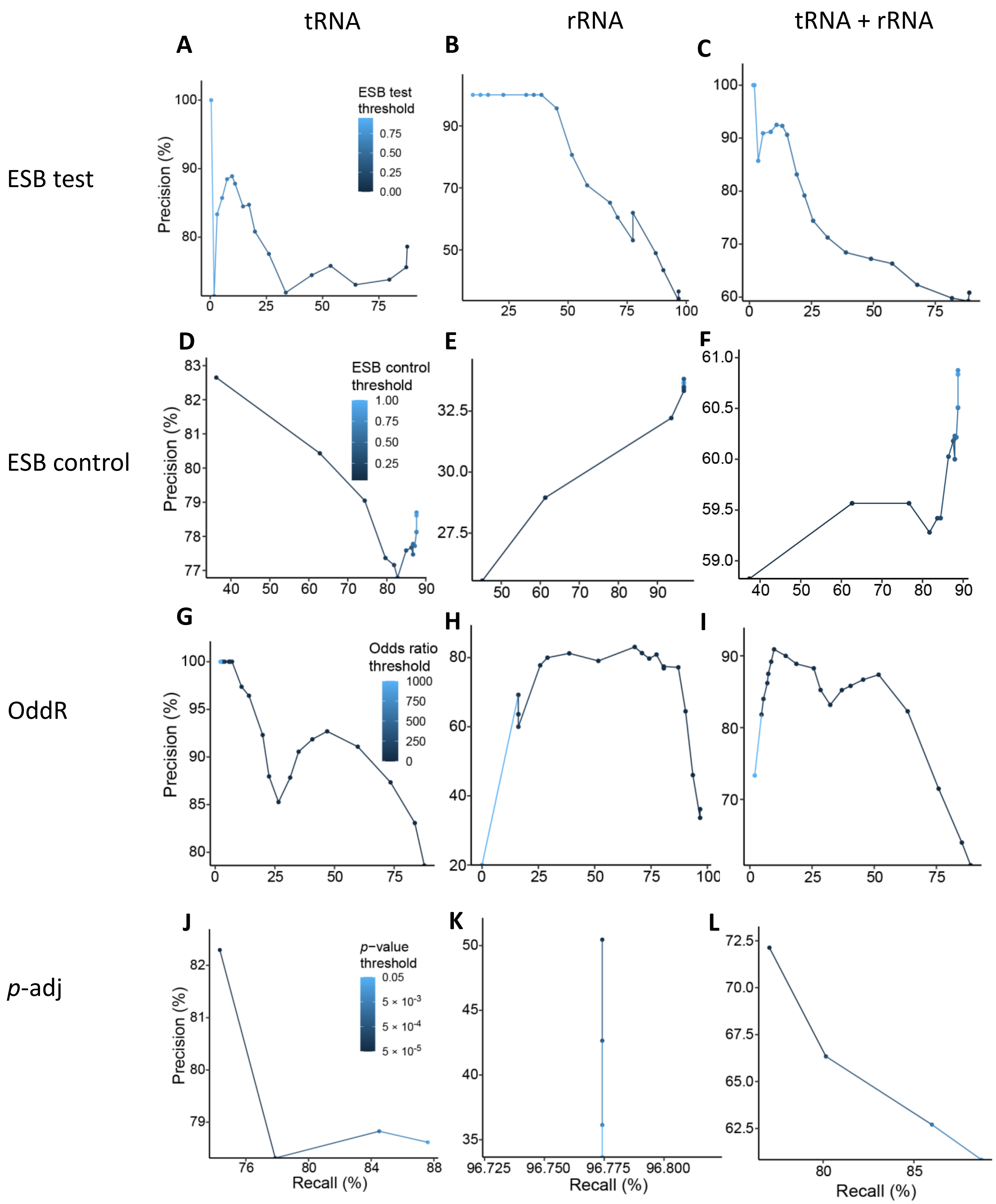

tRNA
rRNA
tRNA + rRNA
ESB test
ESB control
OddR
p-adj
A
B
C
D
E
F
G
H
I
J
K
L
ESB test threshold
ESB control threshold
Odds ratio threshold
p−value threshold
Precision (%)
Recall (%)

Supplementary Figure 9. Precision and recall of known modification sites in *E. coli* tRNA and rRNA as detected with ELIGOS. Modification sites in the *E. coli* rRNA and tRNA were detected with ELIGOS, then the results were examined using varying thresholds of the (A–C) percent error of specific bases (%ESB) in WT *E. coli* RNA (ESB test), (D–F) %ESB in the IVT control RNA (ESB control), (G–I) odds ratio (ESB test/ESB control) (oddR), and (J–L) adjusted *p*-value (*p*-adj). Left, tRNA only; center, rRNA only; right, tRNA + rRNA.

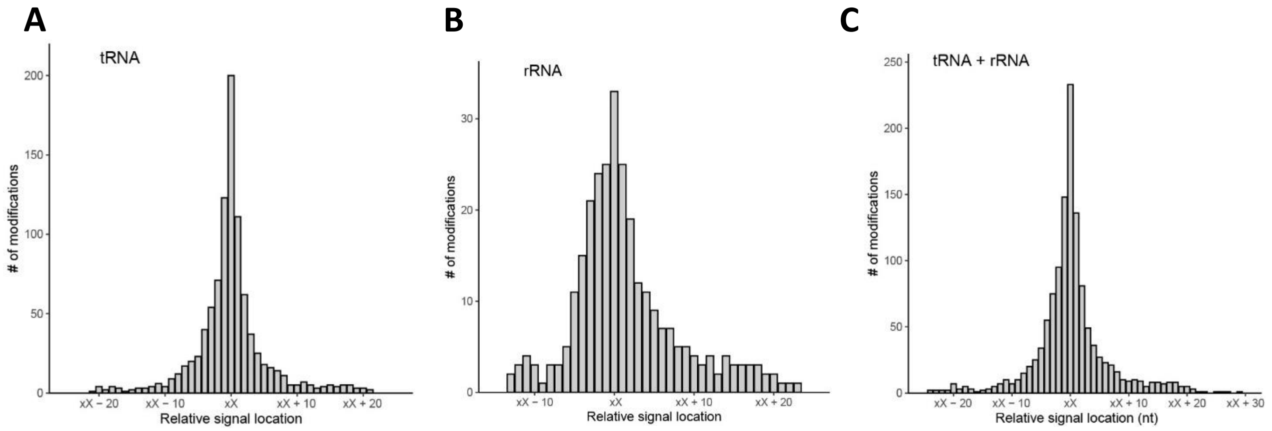

Supplementary Figure 10. Distribution of modification signals around known modified sites. The signals in the direct RNA sequencing data surrounding known modification sites (xX) were examined in the (A) tRNA only, (B) rRNA only, and (C) tRNA and rRNA combined.

Supplementary Table 1. Primers for mutant construction.

Name	Sequence (5' to 3')
DEL_rlmF_for_f1	GGCGCCAAGCTTCTCTGCAGGATCCGCCTGCGGAATTAATGAC
DEL_rlmF_rev_f1	CTGGCGATTGACCGGTTTCTGGGCGGACATAGC
DEL_rlmF_for_f2	CCGCCCAGAAACCGGTCAATCGCCAGCGTTAAAG
DEL_rlmF_rev_f2	GCTAGCGAATTCGTGGATCCAGATGTGACTATCGGGCCGTTGAC
DEL_rlmF_chk_for	AGGCGAATGCAGTTACGTTG
DEL_rlmF_chk_rev	CGGCATGAACACTGGAGATTTG
DEL_rlmJ_for_f1	GGCGCCAAGCTTCTCTGCAGGATCCTACCCAGGTGCTGTATTC
DEL_rlmJ_rev_f1_short	CCGCCAGTTCTTAGTGGCGATAACTGAGCATGG
DEL_rlmJ_for_f2_short	TTATCGCCACTAAGAACTGGCGGTACTGCCAGAC
DEL_rlmJ_rev_f2_short	GCTAGCGAATTCGTGGATCCAGATCATTTCTGGCCGTACATAGC
DEL_rlmJ_chk_for	GGTCGCGATACGCTTTATAC
DEL_rlmJ_chk_rev	TATTCCACGCCCGGAATATC
trmM Fw v2	TGTATGGATATCGCTTCTCGGGTATTGTTCAATTAACCCTCACTAAAGGGCG
trmM Rv v2	AAAGCCTGGGTCAGCGCCGTATACGCTTCGTAATACGACTCACTATAGGGCTC
c fw trmM	TTCGTCGAGTTCAAGTTCG
c Rv trmM	GATTATCCGGAACTGCTCAC

Supplementary Table 2. Primers for reverse transcription quantitative PCR (RT-qPCR).

Name	Sequence (5' to 3')
RT-qPCR recA Fw	CGGTTCGCTTTCACTGGATATCG
RT-qPCR recA rv	CCTGCAGCGTCAGCGTGGT
23S Fw	TAACCGGCGATTTCCGAATG
23S rv	CCAGACGCTTCCACTAACAC
16S Fw	CAGGCGGTTTGTTAAGTCAG
16S rv	CTTCGCCACCGGTATTCCTC
qPCRF for SELECT	ATGCAGCGACTCAGCCTCTG
qPCRR for SELECT	TAGCCAGTACCGTAGTGCGTG

Supplementary Table 3. Mass transitions in dynamic multiple reaction monitoring mode with tandem quadrupole mass spectrometry (MS).

Compound name	Precursor ion	MS1 Res.	Product ion	MS2 Res.	Ret time (min)	Delta Ret time	Fragmentor	Collision energy
**A**	268,1	Wide	136	Unit	5,3	1	180	21
**A SILIS**	283	Wide	146	Unit	5,3	1	180	21
**C**	244,1	Wide	112	Unit	1,8	1	175	13
**C SILIS**	256	Wide	119	Unit	1,8	1	175	13
**Cm**	258,1	Wide	112	Unit	3,8	4	180	9
**Cm SILIS**	271	Wide	119	Unit	3,8	4	180	9
**D**	247,1	Wide	115	Unit	1,7	1	70	5
**D SILIS**	258	Wide	121	Unit	1,7	1	70	5
**G**	284,1	Wide	152	Unit	4,2	1	130	17
**G SILIS**	299	Wide	162	Unit	4,2	1	130	17
**Gm**	298,1	Wide	152	Unit	4,9	1	100	9
**Gm SILIS**	314	Wide	162	Unit	4,9	1	100	9
**m^1G**	298,1	Wide	166	Unit	4,9	1	105	13
**m^1G SILIS**	314	Wide	177	Unit	4,9	1	105	13
**m^2G**	298,1	Wide	166	Unit	4,89	0,6	95	17
**m^2G SILIS**	314	Wide	177	Unit	5,1	0,6	95	17
**m^3U**	259,1	Wide	127	Unit	4,7	2	75	9
**m^4Cm**	272	Wide	126	Unit	4,63	1	80	12
**m^5C**	258,1	Wide	126	Unit	3,6	1	185	13
**m^5C SILIS**	271	Wide	134	Unit	3,6	1	185	13
**m^5U**	259,1	Wide	127	Unit	4,3	2	95	9
**m^5U SILIS**	271	Wide	134	Unit	4,3	2	95	9
**$m^{66}A$**	296	Wide	164	Unit	7,2	1	130	21
**$m^{66}A$ SILIS**	313	Wide	176	Unit	7,2	1	130	21
**m^6A**	282,1	Wide	150	Unit	6,7	1	125	17
**m^6A SILIS**	298	Wide	161	Unit	6,7	1	125	17
**m^7G**	298,1	Wide	166	Unit	3,5	2	100	13
**m^7G SILIS**	314	Wide	177	Unit	3,5	2	100	13
**ms^2i^6A**	382	Wide	250	Unit	7	5	110	21
**ms^2i^6A SILIS**	403	Wide	266	Unit	7	5	110	21
**t^6A**	413,1	Wide	281,1	Unit	6	1	130	9
**t^6A SILIS**	434	Wide	297	Unit	6	1	130	9
**U**	245,1	Unit	113	Unit	3	1	95	5
**U SILIS**	256	Unit	119	Unit	3	1	95	5
**Um**	259,2	Wide	113	Unit	4,6	0,8	96	8
**Um SILIS**	271,1	Wide	119	Unit	4,6	0,8	96	8
**Ψ**	245,1	Wide	209	Unit	1,7	1	90	5
**Ψ SILIS**	256	Wide	220	Unit	1,7	1	90	5

Ret, retention; Res, resolution.

Supplementary Table 4. Primers for used for cloning.

Name	Sequence (5' to 3')
RlmJ GA Fw	TTAACTTTAAGAAGGAGATATACCATGCTCAGTTATCGCCACAGC
RlmJ GA Rv	CTCGAGTGCGGCCGCAAGCTTCAACTCCGGCACGATCCAGCTTAC
pET 28 fw	TTGAAGCTTGCGGCCG
pET 28 rv	GGTATATCTCCTTCTTAAAGTTAAACAA
rlmF for NcoI	CATGCCATGGCAATGTCCGCCCAGAAACC
rlmF rev HIS HindIII	CGGGAAGCTTCTAGTGATGGTGGTGATGGTGTCCACCTCCACGCTGGCGA TTGACAAAACGGC

Supplementary Table 5. Primers for *in vitro* transcription of selected genes.

Name	Sequence (5' to 3')
A1618	TCTACCTGACCACCTGTGTCGGTTTGGGGTCTATAGTGAGTCGTATTA
A2030	GTACACTGCATCTTCACAGCGAGTTCCCTATAGTGAGTCGTATTA
T7-cas3 Fw	TAATACGACTCACTATAGGCTAGCTCATCCAGAACAA
cas3 rv	TACGCTGAACGATCGTC
T7-cysJ Fw	TAATACGACTCACTATAGTCTCTTCTGTTTTATGGGCG
cysJ rv	CAGCAGGCTGCTGATT
T7-rpoH Fw	TAATACGACTCACTATAGGATGTTGTCGGCTGAC
rpoH rv	GAACGGCGAAGGAGAC
T7-phoP Fw	TAATACGACTCACTATAGGCAGGACAAAGTCGAAGTATT
Phop rv	TTTGCCATTATTGCGTATC
T7-uvrY Fw	TAATACGACTCACTATAGACGTTCTACTTGTTGATGACC
uvrY rv	ACGGTAAGCATGATGATTTTG
T7-ppx Fw	TAATACGACTCACTATAGCACATGGTCATAGCCCG
ppx rv	ATACAGACGCTGGCAGG
T7-mazE Fw	TAATACGACTCACTATAGATGATCCACAGTAGCGTAAA
mazE rv	ACCAGTTCAGCAAGCGT
T7-dnaK Fw	TAATACGACTCACTATAGATCGCGGTTTATGACCT
dnak rv	GAGCGGAAGACAGTTCG

Supplementary Table 6. Primers for single-base elongation- and ligation-based quantitative PCR amplification method (SELECT) to detect N^6-methyladenosine (m^6A) modifications.

**Name**	**Sequence (5' to 3')**
23S 2030 control down	5PHOS/TCAATTTCACTGAGTCTCGcagaggctgagtcgctgcat
23S 2030 control up	tagccagtaccgtagtgcgtgGCATCTTCACAGCGAG
23S 2030 m6A down	5PHOS/CACAGCGAGTTCAATTTcagaggctgagtcgctgcat
23S 2030 m6A up	tagccagtaccgtagtgcgtgCGGGTACACTGCATCT
23S 1618 control down	5PHOS/GCTTAGAGGCTTTTCCTGcagaggctgagtcgctgcat
23S 1618 control up	tagccagtaccgtagtgcgtgCGATTTGATGTTACCTGA
23S 1618 m6A down	5PHOS/GTGTCGGTTTGGGGTAcagaggctgagtcgctgcat
23S 1618 m6A up	tagccagtaccgtagtgcgtgATTCTCTACCTGACCACC
cspE 7 m6a down	5PHOS/AGACATCAAAATTACCTTTAcagaggctgagtcgctgcat
cspE 7 m6a up	tagccagtaccgtagtgcgtgCTTAACGTTACCTTTAATCT
cspE control down	5PHOS/GAAACCGAATCCTTTGGACcagaggctgagtcgctgcat
cspE control up	tagccagtaccgtagtgcgtgTGCTGCCGTCTTCCGGAGTAA
dnak 844 m6A down	5PHOS/AACGTCGGTCTGCTGAGCcagaggctgagtcgctgcat
dnak 844 m6A up	tagccagtaccgtagtgcgtgGCAGTGATGTATGGCAGGT
dnak control down	5PHOS/GTGTTTCGGACCGGTCGcagaggctgagtcgctgcat
dnak control up	tagccagtaccgtagtgcgtgACGAGTCACTTTGATGTTCA
groL 331 m6A down	5PHOS/GCCCGCAGCAACAGCTTTcagaggctgagtcgctgcat
groL 331 m6A up	tagccagtaccgtagtgcgtgTTTCAGGTCCATCGGGTTC
groL control down	5PHOS/CGGGTTCATGCCCGCAGcagaggctgagtcgctgcat
groL control up	tagccagtaccgtagtgcgtgCGATACCACGTTTCAGGTCCA
ompC 167 m6A down	5PHOS/AGGTCTGGTCGCCATCTACAcagaggctgagtcgctgcat
ompC 167 m6A up	tagccagtaccgtagtgcgtgCTTTGAAGCCAAGACGCATG
ompC control down	5PHOS/GGTCAGTAACCTGAGTTTCAcagaggctgagtcgctgcat
ompC control up	tagccagtaccgtagtgcgtgGGCCGTAACCGGTCAGC
rpmF 9 m6A down	5PHOS/ACGGCCATGGACCTTACTCcagaggctgagtcgctgcat
rpmF 9 m6A up	tagccagtaccgtagtgcgtgCGGGTTGGTTTATTCTGTTG
rpmF control down	5PHOS/ACAGACAGCTGGTGACTCcagaggctgagtcgctgcat
rpmF control up	tagccagtaccgtagtgcgtgTTTTCACCAGAAGTTTTGTC
rpoA 250 m6A down	5PHOS/GAGCAGGATTTCCAGGATATcagaggctgagtcgctgcat
rpoA 250 m6A up	tagccagtaccgtagtgcgtgCGCCAGCCCTTTCAGGT
rpoA control down	5PHOS/AACTTCATCTTTGCCCTcagaggctgagtcgctgcat
rpoA control up	tagccagtaccgtagtgcgtgAGATTTATTCAAGGTAAGAA
rpsA 1088 m6A down	5PHOS/TGTTGTGGGTTTCCGCGAACcagaggctgagtcgctgcat
rpsA 1088 m6A up	tagccagtaccgtagtgcgtgTACCTTCAACACGGTCGCCC
rpsA control down	5PHOS/GTCAGACAGGTGAACCAGGcagaggctgagtcgctgcat
rpsA control up	tagccagtaccgtagtgcgtgCTGCAACGTTCCAGGAGA
secY control down	5PHOS/AAGCCACCTTTGGCACTTTGAcagaggctgagtcgctgcat
secY control up	tagccagtaccgtagtgcgtgGTCTGCGTTTCAGCTCGCC
secY23 m6A down	5PHOS/CTAATCCCGGTTGTTTAGCCcagaggctgagtcgctgcat
secY23 m6A up	tagccagtaccgtagtgcgtgCCACCTTTGGCACTTTGAAAA

Supplementary Table 7. Primers for *in vitro* RNA transcription from total WT *E. coli* RNA.

**Name**	**primer sequence 5' to 3'**
IVT Fwd Primer PCR	TAATACGACTCACTATAGCGAGGCGGTTTTCTGTTGGTGCTGATATTGCT
IVT Rev Primer PCR	ACTTGCCTGTCGCTCTATCTTC
23S Fw T7	TAATACGACTCACTATA GGTTAAGCGACTAAGCGTACA
23S Rev T7	AAGGTTAAGCCTCACGGTT
16S Fw T7	TAATACGACTCACTATAAATTGAAGAGTTTGATCATGG
16S Rev T7	TAAGGAGGTGATCCAACCG
